# Fraction of MHCII and EpCAM expression characterizes distal lung epithelial cells for alveolar type 2 cell isolation

**DOI:** 10.1186/s12931-017-0635-5

**Published:** 2017-08-07

**Authors:** Koichi Hasegawa, Atsuyasu Sato, Kazuya Tanimura, Kiyoshi Uemasu, Yoko Hamakawa, Yoshinori Fuseya, Susumu Sato, Shigeo Muro, Toyohiro Hirai

**Affiliations:** 1 Department of Respiratory Medicine, Kyoto University Graduate School of Medicine, 54 Kawahara Shogoin Sakyo, Kyoto, 606-8507 Japan; 2 Pulmonary Medicine, Kishiwada City Hospital, 1001 Gakuhara Kishiwada, Osaka, 596-8501 Japan; 3 Pulmonary Medicine, Otsu City Hospital, 2 Chome-9-9 Motomiya Otsu, Shiga, 520-0804 Japan

**Keywords:** Alveolar type 2 cell, EpCAM, MCHII, Cell isolation

## Abstract

**Backgound:**

Alveolar type 2 (AT2) cells play important roles in maintaining adult lung homeostasis. AT2 cells isolated from the lung have revealed the cell-specific functions of AT2 cells. Comprehensive molecular and transcriptional profiling of purified AT2 cells would be helpful for elucidating the underlying mechanisms of their cell-specific functions. To enable the further purification of AT2 cells, we aimed to discriminate AT2 cells from non-AT2 lung epithelial cells based on surface antigen expression via fluorescence activated cell sorting (FACS).

**Methods:**

Single-cell suspensions obtained from enzymatically digested murine lungs were labeled for surface antigens (CD45/CD31/epithelial cell adhesion molecule (EpCAM)/ major histocompatibility complex class II (MHCII)) and for pro-surfactant protein C (proSP-C), followed by FACS analysis for surface antigen expression on AT2 cells. AT2 cells were sorted, and purity was evaluated by immunofluorescence and FACS. This newly developed strategy for AT2 cell isolation was validated in different strains and ages of mice, as well as in a lung injury model.

**Results:**

FACS analysis revealed that EpCAM^+^ epithelial cells existed in 3 subpopulations based on EpCAM and MHCII expression: EpCAM^med^MHCII^+^ cells (Population1:P1), EpCAM^hi^MHCII^−^ cells (P2), and EpCAM^low^MHCII^−^ cells (P3). proSP-C^+^ cells were enriched in P1 cells, and the purity values of the sorted AT2 cells in P1 were 99.0% by immunofluorescence analysis and 98.0% by FACS analysis. P2 cells were mainly composed of ciliated cells and P3 cells were composed of AT1 cells, respectively, based on the gene expression analysis and immunofluorescence. EpCAM and MHCII expression levels were not significantly altered in different strains or ages of mice or following lipopolysaccharide (LPS)-induced lung injury.

**Conclusions:**

We successfully classified murine distal lung epithelial cells based on EpCAM and MHCII expression. The discrimination of AT2 cells from non-AT2 epithelial cells resulted in the isolation of pure AT2 cells. Highly pure AT2 cells will provide accurate and deeper insights into the cell-specific mechanisms of alveolar homeostasis.

**Electronic supplementary material:**

The online version of this article (doi:10.1186/s12931-017-0635-5) contains supplementary material, which is available to authorized users.

## Background

The lung is a vital and complex organ, wherein multiple types of cells are carefully arranged to facilitate gas exchange between the outside environment and the blood. The alveoli are in the most distal part of the lung for gas exchange and are covered with two types of epithelial cells: alveolar type 1 (AT1) cells and alveolar type 2 (AT2) cells [1].

AT2 cells play a central role in maintaining alveolar homeostasis by producing pulmonary surfactant, which regulates the surface tension of alveoli and contributes to host defenses [2, 3]. AT2 cells also have a role in regenerating distal lung epithelial cells through their progenitor function [4, 5]. Lineage-tracing models recently demonstrated that AT2 cells proliferate and differentiate into alveolar type 1 (AT1) cells not only after lung injury but also under non-stressed conditions [6–8]. In turn, the loss of alveolar homeostasis could lead to lung disease development. In fact, damage to or dysregulation of AT2 cells has been implicated in various lung diseases, such as chronic obstructive pulmonary disease (COPD) [9], lung adenocarcinoma [10], and pulmonary fibrosis [11]. As evidence of the roles that AT2 cells play in alveolar homeostasis accumulates, elucidating mechanisms underlying their functions in normal lungs and their possible dysfunction in diseases is becoming more important.

To explore the functions of AT2 cells further, various studies have attempted to characterize AT2 cells isolated from digested whole lung cells [12–15]. Given the availability of novel, high-throughput technologies such as comprehensive transcriptional profiling, highly purified AT2 cells from the lung should offer deeper insights into cell-specific responses to various stimuli in the alveolar region. Negative selection for AT2 cell isolation has been reported previously [13, 14]. A certain level of contamination by non-AT2 cell populations is assumed from the reported purity for this selection; however, this contamination was not characterized in detail. More recently, positive selection using pan-epithelial cell markers such as epithelial cell adhesion molecule (EpCAM) or E-cadherin has increased the purity of this cell population [16, 17] without discriminating between AT2 cells and non-AT2 lung epithelial cells. In pursuit of further purification, identifying AT2 cell surface markers that can separate AT2 cells from non-AT2 lung epithelial cells is desirable.

We hypothesized that major histocompatibility complex class II (MHCII) is an AT2 cell surface marker. While MHCII is important in adaptive immunity in antigen presenting cells, it is also expressed on the surface of AT2 cells in both humans [18] and rodents [19, 20]. Although the role of MHCII in AT2 cells remains to be fully elucidated, recent studies have suggested that it modulates immunoresponses in the lung [20, 21].

In the present study, we fully characterized MHCII expression on murine AT2 cells. Then, we classified distal lung epithelial cells into subpopulations based on EpCAM and MHCII expression levels; one of these populations was found to be enriched by AT2 cells. Finally, we developed a new fluorescence activated cell sorting (FACS)-based strategy for AT2 cell isolation that is widely applicable in the study of AT2 cells.

## Methods

The study protocols were approved by the Animal Research Committee of Kyoto University (ID: MedKyo 43,125).

### Animals

Nine-week-old C57BL/6 J mice (Charles River, Japan), BALB/c mice (SLC, Japan), FVB/N mice (CLEA, Japan), and A/J mice (SLC, Japan) were purchased for use in this study. Double-transgenic Scgb1a1-rtTA (Line 1)/(tetO)^7^CMV-Cre mice (a gift from Dr. Machiko Ikegami and Jeffrey A. Whitsett) [22] were bred with ROSA^mT/mG^ mice (the Jackson Laboratory) to generate triple-transgenic Scgb1a1-rtTA/(tetO)^7^CMV-Cre/ROSA^mT/mG^ mice. Doxycycline (600 ppm) was added to the chow starting from 5 weeks old to 8 weeks old to activate cre-mediated recombination in the triple-transgenic mice.

### The preparation of single-cell suspensions of murine lung

An overview flowchart of cell isolation protocol with estimated time and the yield of cells are depicted in Additional file 1: Figure S1. Single lung cells were obtained using Corti’s protocol [13] with some modifications. Briefly, mice were anesthetized via intraperitoneal injection of pentobarbital with 10 U of heparin. The abdominal cavity was opened, and mice were exsanguinated via transection of the abdominal aorta. The trachea was exposed and intubated with a 20-gauge catheter (Terumo, Tokyo, Japan). The thoracic cavity was opened, and the lungs were perfused with 10 mL of cold 0.9% saline from the right ventricle. In total, 1.5 mL of dispase solution (Corning, Corning, NY) was instilled into the lungs from the intubated catheter, followed by 0.2 mL of 1% low melting point (LMP) agarose (Wako Pure Chemical Industries, Osaka, Japan). The lungs were immediately covered with ice and incubated for 2 min. After incubation, the lungs were isolated from the thoracic cavity and incubated for 45 min in 5 mL of Hank’s Balanced Salt Solution (HBSS) (Wako Pure Chemical Industries) at room temperature (RT). Then, the lungs were minced using scissors and a transfer pipette in Dulbecco’s Modified Eagle Medium (DMEM) (Nacalai Tesque, Kyoto, Japan) containing 25 mM 4-(2-hydroxyethyl)-1-piperazineethanesulfonic acid (HEPES) (Life Technologies, Gaithersburg, MD), antibiotics, antimycotic solution (Life Technologies). After mincing, 100 μL of 1 mg/mL DNase I (Sigma-Aldrich, St. Louis, MO) was added to the sample and incubated for 4 min at RT. The cells were sequentially filtered through 70-μm and 40-μm filters (BD Biosciences, San Jose, CA). Then, the cell suspension was centrifuged for 7 min at 340×g and washed once with Phosphate buffered saline (PBS) (Nacalai Tesque). Next, the cell pellet was suspended with 3 mL of lysis buffer (eBioscience, San Diego, CA), and incubated for 2 min at RT, followed by washing with PBS. The cell pellet was resuspended in PBS containing 3% fetal bovine serum (FBS) for cell isolation or in staining buffer (PBS/0.1% bovine serum albumin (BSA)/0.02% NaN_3_/0.5 mM ethylenediaminetetraacetic acid (EDTA)) for cell analysis. The average number of cells in the single-cell lung suspensions was 9.0 ± 0.9 × 10^6^ /lung, and the viability was 83.0 ± 1.2% (*n* = 8). For the 3-week-old mice, 22-gauge catheters were used for the tracheal intubation, and the instilled volume of dispase solution and 1% LMP agarose were reduced to 1 mL and 0.1 mL, respectively.

### Flow cytometric analysis and sorting of distal lung epithelial cells

A list of antibodies used for flow cytometry is shown in Additional file 2: Table S1. For cell-surface antigen staining, the samples were incubated with antibodies for 20 min at 4 °C, washed and resuspended in PBS containing 3% FBS. To exclude dead cells, 7-amino actinomycin D (7-AAD, BD Biosciences) was added to the samples before sorting. We sorted live, single cells using a FACSAria III Cell Sorter with FACSDiva ver. 8.0.1 (BD Biosciences). Sorted cells were collected in DMEM containing 10% FBS, antibiotics, antimycotic solution, and 25 mM HEPES for further analyses. For proSP-C staining, single cell suspension of the lung or the sorted cells were incubated with fixable viability dye eFluor780 (eBioscience) and surface antigens, fixed, and permeabilized with fixation and permeabilization buffer (eBioscience) according to the manufacturer’s instructions. Permeabilized cells were incubated with anti-mouse proSP-C antibody or rabbit IgG (Santa Cruz Biotechnology, Santa Cruz, CA) and labeled with PE-conjugated F(ab’)2 donkey anti-rabbit IgG (1:200; eBioscience). Fixed cells were analyzed using a BD LSR Fortessa (BD Biosciences). Appropriate isotype control samples were utilized for all FACS analyses. The data were analyzed using FlowJo software (ver. 7.6.5, Tree Star, San Carlos, CA).

### Lipopolysaccharide (LPS)-induced lung injury

Mice were anesthetized with isoflurane and hung upright at a 45-degree angle. LPS from *E. coli* (Sigma-Aldrich, St. Louis, MO) (1 mg/kg body weight in 100 μL of PBS) or PBS (control) was aspirated intratracheally as reported previously [23]. The mice were sacrificed at 24 h after intratracheal instillation for further analyses.

Methods for immunofluorescence and RT-PCR analyses are provided in the online Data Supplement.

### Statistical analysis

The values are expressed as the means ± SEM. Statistical analyses were performed using JMP ver. 10 (SAS Institute, Cary, NC). Comparisons between two groups were performed using the Wilcoxon rank sum test.

## Results

### MHCII expression in AT2 cells

**Fig. 1:**
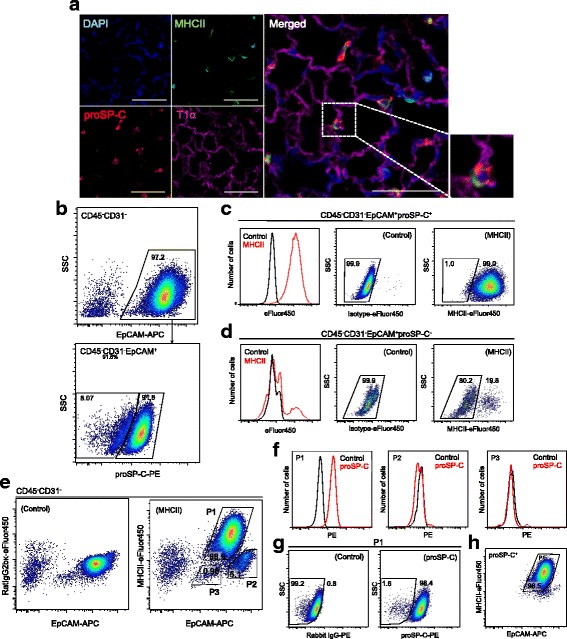
MHCII expression in murine AT2 cells. **a** Immunofluorescence analysis of cells from 9-wk.-old mice shows MHCII expression on proSP-C^+^ AT2 cells. Note that AT1 cells are negative for MHCII expression. Scale bars, 50 μm. **b** Representative FACS plots show that EpCAM^+^ cells were identified among CD45^−^CD31^−^ cells *(top)* and analyzed for proSP-C expression *(bottom)*. **c** MHCII expression in proSP-C^+^ cells. The histogram shows that proSP-C^+^ cells express MHCII *(left)*. Compared to control *(middle)*, 99.0% of proSP-C+ cells are positive for MHCII expression *(right)*. **d** MHCII expression in proSP-C^−^ cells. Histogram *(left)* and FACS plots *(middle and right)* show that the majority of proSP-C^−^ cells are negative for MHCII expression. **e** Representative FACS plot shows that EpCAM^+^ cells are classified into 3 subpopulations (P1, P2, and P3) based on EpCAM and MHCII expression *(right)* compared to control *(left)*. **f** EpCAM^med^MHCII^+^ cells (P1) *(left)* are positive for proSP-C expression. EpCAM^hi^MHC^−^ cells (P2) *(middle)* and EpCAM^low^MHC^−^ cells (P3) *(right)* are negative for proSP-C expression. **g** Representative FACS plot shows that almost all P1 cells are positive for proSP-C expression *(right)* compared to controls *(left)*. **h** EpCAM^+^proSP-C^+^ cells were back-gated to the plot of EpCAM and MHCII. Note that most proSP-C^+^ cells were in the P1 gate. AT1 cells, alveolar type 1 cells; AT2 cells, alveolar type 2 cells; EpCAM, epithelial cell adhesion molecule; MHCII, major histocompatibility complex II; proSP-C, pro-surfactant protein C; DAPI, 4′,6-diamidino-2-phenylindole; SSC, side scatter; PE, phycoerythrin; APC, allophycocyanin

To demonstrate the localization of MHCII in adult murine lungs, we analyzed MHCII expression by immunofluorescence. As shown in Fig. 1a, proSP-C^+^ AT2 cells also expressed MHCII, while AT1 cells were negative for MHCII. In the alveoli, alveolar macrophages were also positive for MHCII expression. All proSP-C^+^ cells were positive for MHCII expression.

To investigate MHCII expression in AT2 cells further, we performed FACS analysis of component cells of murine lungs. Single-cell suspensions obtained from enzymatically digested lungs were stained for surface antigens, fixed, permeabilized, and then stained for proSP-C. Using FACS analysis, CD45^−^CD31^−^EpCAM^+^ cells (henceforth, EpCAM^+^ cells) were analyzed for proSP-C expression (Fig. 1b). Among EpCAM^+^ cells, 90.4% ± 1.7% were positive for proSP-C expression, and almost all proSP-C^+^ cells expressed MHCII (99.0 ± 0.2%) (Fig. 1c). In contrast, the majority of proSP-C^−^ EpCAM^+^ cells were negative for MHCII expression (Fig. 1d). This observation suggests that EpCAM^+^ cells from enzymatically digested murine lungs primarily consist of AT2 cells but also contain a substantial amount of proSP-C^−^ epithelial cells. Thus, MHCII could be a useful surface marker for classifying lung epithelial cells to identify AT2 cells.

In the two-dimensional plot of EpCAM and MHCII, EpCAM^+^ cells were classified into 3 different subpopulations based on EpCAM and MHCII expression: EpCAM^med^MHCII^+^ cells (Population 1; P1 cells), EpCAM^hi^MHCII^−^ cells (P2 cells), and EpCAM^low^MHCII^−^ cells (P3 cells) (Fig. 1e). Most P1 cells were positive for proSP-C expression (97.8 ± 0.4%), while P2 and P3 cells were negative for proSP-C expression (Fig. 1f and g). To evaluate the efficiency of the gating strategy for AT2 cell identification, proSP-C^+^ cells were back-gated in the plot of EpCAM and MHCII, demonstrating that 97.6 ± 0.3% of the cells were in the P1 gate (Fig. 1h).

### Isolation of AT2 cells based on EpCAM and MHCII expression

**Fig. 2:**
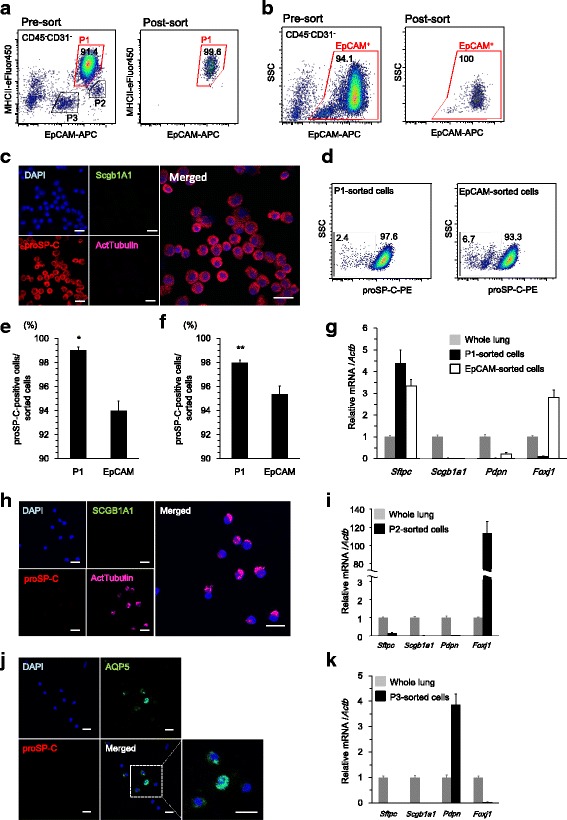
Lung epithelial cell isolation. Live single lung cells were stained for CD45, CD31, EpCAM, and MHCII. CD45^−^CD31^−^ cells were analyzed by cell sorting. **a** CD45^−^CD31^−^ cells were analyzed for EpCAM and MHCII expression to sort P1 cells *(left).* The sorted cells were reanalyzed *(right).* **b** CD45^−^CD31^−^ cells were analyzed for EpCAM expression and SSC (side scatter) to sort EpCAM^+^ cells *(left*). The sorted cells were reanalyzed *(right)*. **c** Immunofluorescence analysis of cytospin preparations of sorted P1 cells. Almost all cells were positive for proSP-C expression. Scale bar, 20 μm. **d** FACS analysis of proSP-C expression in sorted P1 *(left)* or EpCAM^+^ cells *(right)*. **e** The proportion of proSP-C^+^ cells analyzed by immunofluorescence among sorted P1 and EpCAM^+^ cells are shown (*n* = 3/group). *p < 0.05. **f** The proportion of proSP-C^+^ cells analyzed by FACS among sorted P1 and EpCAM^+^ cells are shown (*n* = 5/group). **p < 0.01. **f** mRNA expression in sorted P1 and EpCAM^+^ cells relative to that in whole lung cells (whole lung expression = 1). Note that sorted EpCAM^+^ cells have higher *Foxj1* expression compared to sorted P1 cells, although both cells exhibit high *Sftpc* expression (*n* = 3/group). **h** Immunofluorescence analysis of cytospin preparations of sorted P2 cells demonstrating that almost all cells were positive for acetylated tubulin expression. Scale bars, 20 μm. **i** mRNA expression in sorted P2 cells relative to that in whole lung cells (whole lung expression = 1). *Foxj1* is highly expressed (*n* = 3/group). **j** Immunofluorescence analysis of cytospin preparations of sorted P3 cells. Approximately half of the cells were positive for AQP5 expression. P3 cells were negative for SCGB1A1, proSP-C, acetylated tubulin, and T1α expression. (**k**) mRNA expression in sorted P3 cells relative to that of whole lung cells (whole lung expression = 1). *Pdpn* is highly expressed (*n* = 3/group). EpCAM, epithelial cell adhesion molecule; MHCII, major histocompatibility complex II; proSP-C, pro-surfactant protein C; ActTubulin, acetylated tubulin; SCGB1A1, secretoglobin 1A1; AQP5, aquaporin 5; DAPI, 4′,6-diamidino-2-phenylindole; PE, phycoerythrin; APC, allophycocyanin

During AT2 cell isolation, we evaluated whether P1 cell isolation was superior to EpCAM^+^ cell isolation in terms of purity. Live single cells from enzymatically digested lungs were stained for surface antigens. EpCAM^+^ cells were classified into 3 subpopulations as shown in the fixed cell analysis (P1, P2, and P3) (Fig. 2a and Additional file 3: Figure S2). The average yield of P1 cells was 6.2 ± 0.7 × 10^5^ /lung (*n* = 8), and the viability was 89.0 ± 1.3% when assessed with fixable viability dye staining. EpCAM^+^ cells were also sorted (Fig. 2b), and the purity of both sorted cells was evaluated by immunofluorescence and FACS. The average yield of P2 and P3 cells were 1.8 ± 0.1 × 10^4^ and 2.3 ± 0.2 × 10^4^ /lung, respectively (*n* = 3).

Immunofluorescence analysis of cytospin preparations of sorted cells demonstrated that the proportion of proSP-C^+^ cells in sorted P1 cells was 99.0 ± 0.3%, which was significantly higher than that in sorted EpCAM^+^ cells (94.0 ± 0.8%) (Fig. 2c and e).

For FACS analysis of proSP-C expression, sorted cells were fixed, permeabilized and stained for proSP-C. The purity of sorted P1 cells was 98.0 ± 0.2%, whereas that of sorted EpCAM^+^ cells was 95.4 ± 1.5% (Fig. 2d and f), demonstrating again that the purity of P1 cells was significantly higher.

To characterize P1 and EpCAM^+^ cells sorted by surface markers, we performed mRNA expression analysis to confirm cell specific gene expression. *Sftpc* (*Surfactant Protein C*, AT2 cell specific surfactant protein) expression levels were 4.4-fold higher in P1 cells compared to whole lung cells, while the expression of other epithelial lineage markers including *Scgb1a1* (*Secretoglobin Family 1A Member 1*, club cell specific protein), *Pdpn* (*Podoplanin*, AT1 cell specific protein among lung epithelial cells), and *Foxj1* (*Forkhead Box J1*, ciliated cell specific nuclear protein) was minimal (Fig. 2g). In contrast, sorted EpCAM^+^ cells displayed *Pdpn* and *Foxj1* expression.

To characterize P2 and P3 cells, we sorted these subpopulations and performed immunofluorescence and mRNA expression analyses. The majority of P2 cells were positive for acetylated tubulin (92.3 ± 2.0%, *n* = 3), with sparse SCGB1A1^+^ cells and unspecified cells as determined by immunofluorescence analysis (Fig. 2h). *Foxj1* expression in sorted P2 cells was 116-fold higher compared to that in whole lung cells (Fig. 2i), suggesting that P2 cells are enriched with ciliated cells. Approximately half of P3 cells, which were negative for major epithelial cell markers including proSP-C, SCGB1A1, acetylated tubulin, and T1α as determined by immunofluorescence analysis, were positive for AQP5 (Fig. 2j). mRNA analysis revealed 3.9-fold higher *Pdpn* expression (Fig. 2k), suggesting that AT1 cells are the major cell type among P3 cells.

### Validation of the gating strategy using AT2 cells with intrinsic GFP expression

**Fig. 3:**
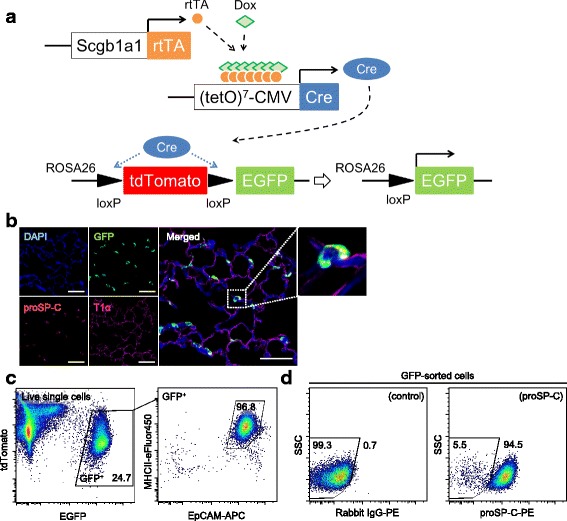
Validation of the gating strategy using AT2 cells with intrinsic GFP expression. **a** Scgb1a1-rtTA/(tetO)^7^CMV-Cre mice were bred with ROSA^mTmG^ mice to generate triple-transgenic mice that express EGFP in AT2 cells following cre-mediated recombination. **b** Immunofluorescence analysis of the lung from recombinant Scgb1a1-rtTA/(tetO)^7^CMV-Cre-mTmG mice demonstrated that proSP-C^+^ AT2 cells express GFP. AT1 cells do not express GFP. Scale bars, 50 μm. **c** GFP^+^ cells of single lung cells *(left)* from recombinant Scgb1a1 (Line 1)-rtTA/(tetO)^7^CMV-Cre-mTmG mice were analyzed in the dot plot of EpCAM and MHCII *(right)*. Almost all GFP^+^ cells were gated with P1 cells. **d** FACS analysis of sorted GFP^+^ cells demonstrates that sorted cells are primarily positive for proSP-C *(right*) compared to controls *(left)*. EGFP, enhanced green fluorescent protein; tdTomato, tandem-dimer Tomato; EpCAM, epithelial cell adhesion molecule; MHCII, major histocompatibility complex II; proSP-C, pro-surfactant protein C; SSC, side scatter; DAPI, 4′,6-diamidino-2-phenylindole

FACS analysis with fluorescent antibody staining is a useful method to explore antigen expression. However, this approach is inevitably accompanied by possible non-specific antibody binding, which we verified using isotype controls. To evaluate the consistency of our gating strategy, we employed AT2 cells that have intrinsic GFP expression. We bred double-transgenic Scgb1a1-rtTA/(tetO)^7^CMV-Cre mice [22] with ROSA^mT/mG^ mice to generate triple-transgenic Scgb1a1-rtTA/(tetO)^7^CMV-Cre/ROSA^mT/mG^ mice (Fig. 3a). In these triple-transgenic mice, most AT2 cells and a subset of club cells display enhanced GFP expression following the addition of doxycycline to the chow (Fig. 3b), starting from 5 weeks old to 8 weeks old to minimize GFP labeling in AT1 cells and ciliated cells due to lung maturation after birth [24].

Using single lung cells obtained from these triple-transgenic mice, we analyzed GFP^+^ cells for EpCAM and MHCII expression and found that 97.4 ± 0.3% (*n* = 4) of GFP^+^ cells were gated with P1 cells (Fig. 3c). Then, we sorted GFP^+^ cells (5.8 ± 0.5 × 10^5^ cells /lung), analyzed proSP-C expression by FACS; the results indicated that most sorted GFP^+^ cells were positive for proSP-C (96.0 ± 0.6%) (Fig. 3d).

### CD74 expression in AT2 cells

**Fig. 4:**
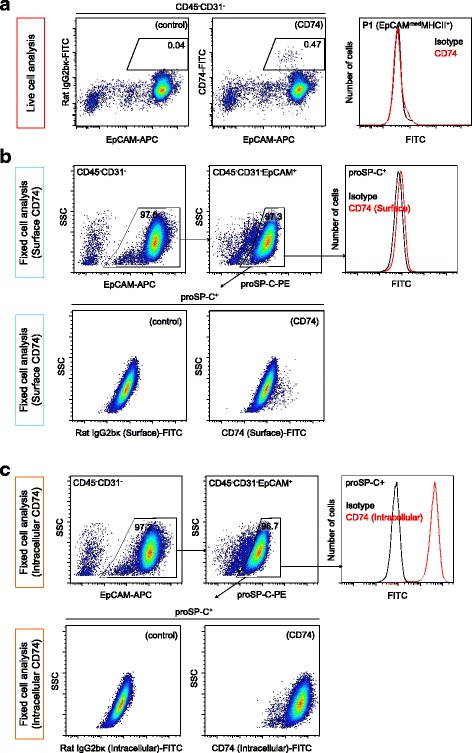
CD74 expression in AT2 cells. **a** Live single cells were stained for CD74. Compared to controls *(left)*, only a small fraction of EpCAM^+^ cells were positive for CD74 expression *(middle and right)*. **b** CD74 staining was performed before fixation and permeabilization of single lung cells (surface staining). EpCAM^+^ cells were identified *(top, left)*, and proSP-C^+^ cells *(top, middle)* were analyzed for CD74 expression *(top, right)*. proSP-C^+^ cells showed uniform but weak CD74 expression. A small fraction of proSP-C^+^ cells expressed CD74 at higher levels than others *(bottom, right)* when compared to controls *(bottom, left)*. **c** CD74 staining was performed after the fixation and permeabilization of single lung cells (intracellular staining). EpCAM^+^ cells were identified *(top, left)*, and proSP-C^+^ cells *(top, middle)* were analyzed for CD74 expression *(top, right. Bottom).* High CD74 expression was observed *(bottom, right and left)* in proSP-C^+^ cells. EpCAM, epithelial cell adhesion molecule; proSP-C, pro-surfactant protein C; SSC, side scatter; PE, phycoerythrin; APC, allophycocyanin; FITC, fluorescein isothiocyanate

Because CD74 has been reported to be a potential surface marker of AT2 cells [25, 26], we evaluated whether CD74 can be utilized for the positive selection of AT2 cells. Live-cell analysis demonstrated that only a small fraction of EpCAM^+^ cells expressed CD74 (Fig. 4a). We further explored CD74 expression among EpCAM^+^ cells using fixed and permeabilized cells stained for proSP-C. When CD74 staining was performed before cell fixation and permeabilization (surface CD74 analysis), proSP-C^+^ cells were found to express CD74 weakly as a whole, with a small fraction of cells showing higher CD74 expression (Fig. 4b), while proSP-C^−^ cells did not express CD74. In contrast, when CD74 staining was performed after fixation and permeabilization (intracellular CD74 analysis), proSP-C^+^ cells displayed much higher CD74 expression compared with isotype controls (Fig. 4c). These observations suggest that epithelial CD74 expression is limited to AT2 cells, although CD74 is not suitable for the positive selection of AT2 cells due to its predominantly intracellular expression. Although why most of the epithelial cells were negative for CD74 in the live-cell analysis is unclear, surface CD74 may be unstable and may be transformed into a soluble form [27] without cell fixation.

### The applicability of the AT2 isolation strategy to different strains and ages of mice

To investigate the applicability of our newly developed AT2 cell isolation strategy, we evaluated EpCAM, MHCII, and proSP-C expression levels in single lung cells from different strains and ages of mice (Table 1, Additional file 4: Figure S3).

**Table 1:** MHCII expression in AT2 cells and the proportion of AT2 cells in total cells and P1 cells from different strains and ages of mice

Strain	Age	The yield of single cell suspension (×10^6^/ lung)	proSP-C^+^ cells/Live single cells (%)	MHCII^+^ cells/proSP-C^+^ cells (%)	proSP-C^+^ cells/P1 cells (%)
	3-wk.-old	4.3 ± 0.6	25.2 ± 1.7	99.2 ± 0.2	97.2 ± 0.2
C57BL/6 J	9-wk.-old	8.1 ± 0.4	34.5 ± 1.9	99.0 ± 0.2	97.9 ± 0.4
	1-yr.-old	9.7 ± 0.8	31.1 ± 1.2	99.2 ± 0.2	97.4 ± 0.3
BALB/c	9-wk.-old	8.2 ± 0.4	32.0 ± 3.5	99.6 ± 0.0	97.7 ± 0.5
FVB/N	9-wk.-old	8.7 ± 0.7	33.3 ± 0.8	99.1 ± 0.2	97.6 ± 0.4

The values are expressed as the means ± SE (*n* = 3/group). MHCII, major histocompatibility complex class II; proSP-C, pro-surfactant protein C

Mouse MHCII is highly polymorphic [28]. We performed FACS analysis using BALB/c, FVB/N, and A/J mice. In BALB/c and FVB/N mice, EpCAM^+^ cells were similarly classified into 3 subpopulations, and proSP-C^+^ cells were enriched in P1 cells (Table 1) in these mice and in C57BL6/J mice. However, in A/J mice, MHCII expression among proSP-C^+^ cells was negative, resulting in the failed classification of EpCAM^+^ cells, although non-EpCAM^+^ cells expressed MHCII, suggesting that AT2 cells in A/J mice lack MHCII expression.

Next, we evaluated young (3-week-old) and old (1-year-old) mice. In both groups, almost all proSP-C^+^ cells expressed MHCII, and P1 cells identified based on EpCAM and MHCII expression were enriched with proSP-C^+^ cells. These results suggested that MHCII expression does not alter as a function of age or mouse strain, except for A/J mice.

### AT2 cell isolation in an LPS-induced lung injury model

**Fig. 5:**
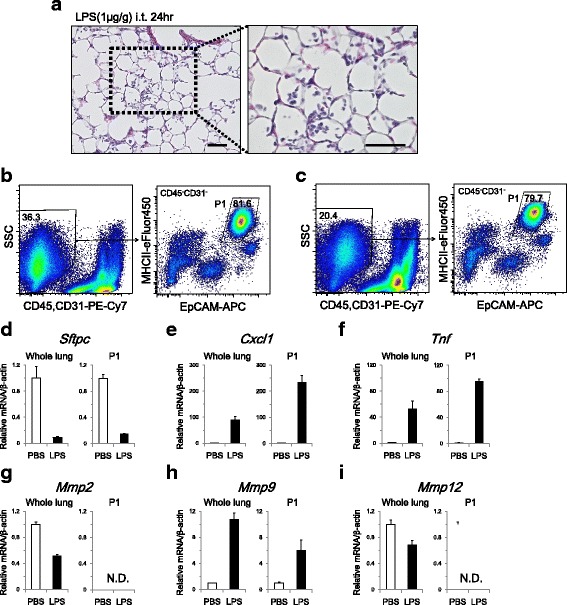
AT2 cell isolation in an LPS-induced lung injury model. **a** Hematoxylin and eosin staining of the lung at 24 h after intratracheal instillation of LPS (1 μg/g body weight). Alveolar spaces were infiltrated with inflammatory cells. Scale bars, 50 μm. **b** Representative FACS plot that identifies CD45^−^CD31^−^ cells *(left)*, which were analyzed for EpCAM and MHCII expression to sort P1 cells *(right)* in control samples. **c** Representative FACS plots of the LPS-induced lung injury model. Compared to the control, the proportion of CD45^−^CD31^−^ cells was lower due to increased numbers of inflammatory cells *(left)*. P1 cells were successfully identified and sorted *(right)*. In both the control and the LPS model, whole-lung samples and sorted P1 cells were analyzed for (**d**) *Sftpc*, (**e**) *Cxcl1*, (**f**) *Tnf*, (**g**) *Mmp2*, (**h**) *Mmp9*, and (**i**) *Mmp12* mRNA expression (*n* = 3/group). In P1 cells, *Cxcl1*, *Tnf*, and *Mmp9* were upregulated following LPS stimulation. *Mmp2* and *Mmp12* were undetected in P1 cells. EpCAM, epithelial cell adhesion molecule; MHCII, major histocompatibility complex class II; PE-Cy7, phycoerithrin-cyanin7; APC, allophycocyanin; LPS, lipopolysaccharide; SSC, side scatter

To evaluate whether we can identify subpopulations of distal lung epithelial cells (P1, P2, and P3) during lung injury, we applied our isolation strategy to an LPS-induced lung injury model. LPS stimulation of the lung induced inflammatory cell infiltration in alveolar spaces (Fig. 5a). Single-cell suspensions of LPS model lungs contained higher numbers of cells (2.2 ± 0.2 × 10^7^ per sample) with the same viability (91.6 ± 1.0% by trypan blue exclusion) as control samples, which were prepared in the same manner (8.3 ± 0.8 × 10^6^ per sample and 89.7 ± 1.2%, respectively). FACS analysis indicated that EpCAM and MHCII expression levels were not significantly altered in distal lung epithelial cells following LPS instillation, which resulted in the successful identification of P1 cells in addition to P2 and P3 cells (Fig. 5b, c, and Additional file 5 **:** Table S2). The yield of P1 cells in LPS model was 4.2 ± 0.3 × 10^5^, which was slightly smaller than that of control samples (5.4 ± 0.3 × 10^5^) due to the lower sorting efficiency in the LPS model, in which the cell suspension contained a greater number of non-AT2 cells. In the LPS model, sorted P1 cells showed 15.6-fold higher *Sftpc* expression compared with whole-lung cells. In P1 cells, *Sftpc* expression was downregulated in response to LPS stimulation (Fig. 5d). *Cxcl1* and *Tnf* were upregulated to higher levels in P1 cells following LPS stimulation (Fig. 5e and f) than those observed in whole-lung cell analyses. In P1 cells, *Mmp9* was upregulated in response to LPS (Fig. 5h), while *Mmp2* and *Mmp12* expression was undetected in both control and LPS model cells (Fig. 5g and i). MMP12 is a metalloprotease that is expressed by macrophages, and the lack of *MMP12* expression in sorted cells further supports the successful depletion of hematopoietic cells from the inflammatory lung injury model.

## Discussion

The present study demonstrated that murine distal lung epithelial cells can be classified into 3 subpopulations (P1, P2, and P3) by FACS analysis of EpCAM and MHCII expression. AT2 cells were highly enriched in the P1 subpopulation (EpCAM^med^MHCII^+^) and were successfully sorted with high purity and viability. P2 (EpCAM^hi^MHCII^−^) contained primarily ciliated cells, and approximately half of P3 (EpCAM^low^MHCII^−^) cells were identified as AT1 cells.

We revealed two important considerations for performing FACS analyses of distal lung epithelial cells. The first consideration is that EpCAM expression levels measured by fluorescence intensity vary widely among EpCAM^+^ cells. The second consideration is that AT2 cells uniformly express MHCII and that the epithelial expression of MHCII is limited to AT2 cells. Together, these two factors enabled the clear classification of distal lung epithelial cells by FACS, leading to highly pure AT2 cell isolation.

EpCAM is known as a pan-epithelial cell marker, yet its relative expression among each type of lung epithelial cell has not been fully investigated. Because EpCAM is a principal marker for the positive selection of AT2 cells, we labeled EpCAM with an APC-conjugated antibody. APC has a high staining index and is superior to other common fluorescent proteins in discriminating positive and negative fractions by FACS analysis (https://www.bdbiosciences.com/documents/lsr_appnote02.pdf). This fluorescence property resulted in the demonstration of a wide range of EpCAM expression levels among distal lung epithelial cells by FACS analysis. In fact, a previous report implied that AT2 cells have lower EpCAM expression compared to bronchiolar cells using an anti-EpCAM antibody conjugated to PE [29], whose staining index is similar to that of APC.

Together with the wide distribution of EpCAM expression levels, the homogenous and specific expression of MHCII in AT2 cells was helpful for discriminating these cells from non-AT2 epithelial cells, particularly bronchiolar cells. In the FACS plot of EpCAM versus MHCII, lung epithelial subpopulations with different EpCAM expression levels were clearly separated based on MHCII expression. Although MHCII is primarily expressed in antigen-presenting cells, AT2 cells also constitutively express MHCII. Recently, the MHCII-dependent immunomodulatory functions of AT2 cells have received increasing attention. Debbabi et al. reported that AT2 cells can process mycobacterial antigens through the MHCII pathway but fail to prime naïve T cells, suggesting a regulatory role for AT2 cells in T cell inflammation [20]. In contrast, Gereke et al. reported that AT2 cells can prime naïve CD4^+^ T cells for self-antigens or exogenous antigens and induce T-cell activation, while upon inflammation, AT2 cells induce regulatory T cells by producing antiproliferative factors such as TGF-β [21].

In the present study, we demonstrated that AT2 cells from A/J mice do not express MHCII; this lack of expression may be because A/J mice show less neutrophil infiltration in response to LPS compared to BL6 mice [30]. Furthermore, A/J mice are highly sensitive to the chemical induction of lung tumors [31]. Although its relevance is unknown, elucidating the relationship between MHCII expression in AT2 cells and lung disease development should be the subject of future research.

We developed a new strategy for isolating AT2 cells using EpCAM and MHCII as positive selection markers. Highly pure AT2 cells can provide accurate and cell-specific information for the study of AT2 cell functions. Even a small fraction of contaminating cells could complicate data interpretation, particularly in transcriptional profiling, as suggested by mRNA analyses of sorted EpCAM^+^ cells in the present study. Although the purity of sorted EpCAM^+^ cells exceeded 95%, *Foxj1* expression derived from contaminating ciliated cells was clearly observed. Previous methods of AT2 cell isolation included negative selection that depletes other cell types, such as hematopoietic cells and endothelial cells from single lung cells [13–15, 26, 32, 33]. Recent strategies have added EpCAM as a positive selection marker, increasing the purity of isolated cells to approximately 95% [16, 17, 34] (Table 2). We further increased the purity of isolated AT2 cells by depleting non-AT2 lung epithelial cells, which primarily consisted of ciliated cells and AT1 cells. Although club cells were abundant in the distal lung tissue, they seemed to be depleted during the process of single lung cell preparation, as suggested by immunofluorescence and mRNA analyses of sorted EpCAM^+^ cells. Analyses of triple-transgenic mice in which AT2 cells are labeled with GFP further support this conclusion. While GFP-labeled club cells were observed via immunofluorescence in the lung tissue, most sorted GFP^+^ cells from the triple-transgenic mice were positive for proSP-C expression.

**Table 2:** Summary of AT2 cell isolation methods

Author	Year	Preparation of single cells	Cell selection	Surface antigens for cell isolation	Cell sorting modality	Purity assessment	Purity	Ref.
Corti et al.	1996	Dispase and LMP agarose	N	CD16/32, CD45	Magnet	PAP staining	92.8%	13
Rice et al.	2002	Dispase and LMP agarose	N	CD45, CD16/32	Antibody-coated plate	PAP staining, SP-C (ICC)	>90%	14
Kim et al.	2005	Dispase and LMP agarose	N	CD31, CD45, Sca-1	FACS	SP-C (IF)	N/A	26
Herold et al.	2006	Dispase and LMP agarose	N	CD16/32, CD45	Magnet	Papanicolaou staining, proSP-C (IF)	>90%	27
Bernice et al.	2008	Dispase and LMP agarose	N	CD45, CD11b, CD11c	Magnet	Papanicolaou staining	71%	15
Marsh et al.	2009	Dispase and LMP agarose	N	CD16/32, CD45	Magnet	proSP-C (FACS)	>80%	28
Teisanu et al.	2011	Elastase	N and P	CD45, CD31, EpCAM, Sca-1	FACS	intrinsic GFP (FACS)	N/A	18
Messier et al.	2012	Dispase and LMP agarose	N and P	CD45, EpCAM	Magnet	SP-A (FACS)	91.1	29
Yamada et al.	2013	Dispase II and collagenase	N and P	CD45, VE-cadherin, EpCAM	FACS	N/A	N/A	19
Yamada et al.	2013	Dispase and LMP agarose	N and P	CD45, EpCAM	Magnet	proSP-C (IF and FACS)	>96%	19
Lee et al.	2013	Dispase and LMP agarose	N and P	CD45, CD31, CD74	FACS	intrinsic GFP (FACS)	91.8%	17
Hasegawa et al.	2015	Dispase and LMP agarose	N and P	CD45, CD31, EpCAM, MHCII	FACS	proSP-C (IF and FACS)	98–99%	

*LMP agarose* low melting point agarose, *N* negative selection, *P* positive selection, *EpCAM* epithelial cell adhesion molecule, *VE-cadherin* vascular endothelial cadherin, *Magnet* magnet-based cell isolation, *FACS* fluorescence-activated cell sorting, *SP-C* surfactant protein-C, *SP-A* surfactant protein-A, *ICC* immunocytochemistry, *IF* immunofluorescence

We extensively validated EpCAM and MHCII expression in lung epithelial cells using different strains and ages of mice, as well as a lung injury model. The stability of EpCAM and MHCII surface expression is crucial to identifying AT2 cells based on surface antigen expression. Except for A/J mouse cells, distal lung epithelial cells were classified in the same manner based on EpCAM and MHCII expression.

In the LPS model, transcriptional analyses revealed the prominent upregulation of *Cxcl1* and *Tnf* in AT2 cells following LPS instillation. Using in situ hybridization, Elizur et al. demonstrated that club cells and AT2 cells express *Cxcl1* following LPS stimulation, while alveolar macrophages are the primary types of cells that express *Tnf* in the distal lung [35]. These authors also reported that TNFα produced by macrophages is important for CXCL1 production by club cells [36], whereas Skerrett et al. reported that *Tnfa* is expressed in bronchiolar epithelial cells following LPS inhalation through NFκB activation, leading to neutrophil recruitment [37]. In contrast with the known role of bronchiolar cells in the immune response, the role of AT2 cells in LPS-induced lung injury has remained largely unknown. Here, we isolated AT2 cells by FACS and investigated specific transcriptional changes in response to LPS stimulation. The upregulation of *Cxcl1* and *Tnf* mRNA expression implies that AT2 cells also have a role in modulating immunological responses by recruiting neutrophils into alveolar spaces, and further investigations are warranted to elucidate the coordinated responses between epithelial cells and hematopoietic cells in the alveolar region.

Whether our isolation strategy is applicable to human AT2 cell isolation is also of interest. For human lung epithelial cell isolation, a previous report demonstrated that EpCAM^+^T1α^−^ cells were enriched with AT2 cells at a purity of 94.0%, as assessed by proSP-C staining, while AT1 cells and club cells were found in the EpCAM^+^T1α^−/lo^ fraction [38]. Interestingly, AT2 cells displayed higher EpCAM expression compared to AT1 cells, as shown in the murine lung. Because the constitutive expression of MHCII in human AT2 cells has been reported previously [39], human AT2 cell isolation using EpCAM and MHCII as positive selection markers is worth considering.

Our study has some limitations. First, the preparation of single cells by protease digestion might affect cell status or surface-antigen expression. Tissue digestion using dispase, which is relatively gentle, has been widely used for many bioassays using AT2 cells. Because our protocol is not appropriate for club cell isolation, optimal digestion methods including proteases would depend on targeted cells in the lung. Second, tissue digestion and cell sorting require a certain amount of time, which might affect the transcriptional status of AT2 cells. To further optimize our methods, we attempted tissue dissociation using a gentle MACS dissociator (Miltenyi Bio-Tech, Bergisch-Gladbach, Germany). This modification shortened the amount of digestion time by 20 min with 3 samples and resulted in approximately 3 times the number of cells in a single-cell suspension without compromising cell viability (>90% with trypan blue exclusion).

Third, the number and purity of the sorted cells were not high enough to analyze the P2 and P3 subpopulations themselves. Although we identified major types of cells that were enriched in these subpopulations, we could not characterize a few of P2 cells and approximately half of P3 cells by immunofluorescence. One possible explanation for this difficulty in characterization is that dispase negatively affected surface-antigen expression [40].

## Conclusion

In conclusion, we demonstrated that distal lung epithelial cells can be classified into 3 subpopulations based on EpCAM and MHCII expression. We successfully discriminated AT2 cells from non-AT2 epithelial cells and sorted AT2 cells with high purity. Highly pure AT2 cells will serve as a powerful tool for molecular and transcriptional analyses and will provide cell-specific information in both normal and diseased lungs.

## Additional files

Additional file 1: Figure S1. An overview flowchart of cell isolation protocol. (PPTX 73 kb)

Additional file 2: Table S1. List of antibodies for flow cytometry. (DOCX 17 kb)

Additional file 3: Figure S2. Gating strategy for isolation of AT2 cells based on EpCAM and MHCII expression. (PPTX 301 kb)

Additional file 4: Figure S3. The applicability of the AT2 isolation strategy to different strains and ages of mice. (PPTX 572 kb)

Additional file 5: Table S2. The yield of single cell suspension and the proportion of each cell population in control and LPS-induced injury mice. (DOCX 14 kb)

## References

[1] Herzog EL, Brody AR, Colby TV, Mason R, Williams MC (2008). Knowns and unknowns of the alveolus. Proc Am Thorac Soc.

[2] Wright JR (2005). Immunoregulatory functions of surfactant proteins. Nat Rev Immunol.

[3] Fehrenbach H (2001). Alveolar epithelial type II cell: defender of the alveolus revisited. Respir Res.

[4] Adamson IY, Bowden DH (1974). The type 2 cell as progenitor of alveolar epithelial regeneration. A cytodynamic study in mice after exposure to oxygen. Lab Investig.

[5] Evans MJ, Cabral LJ, Stephens RJ, Freeman G (1975). Transformation of alveolar type 2 cells to type 1 cells following exposure to NO2. Exp Mol Pathol.

[6] Rock JR, Barkauskas CE, Cronce MJ, Xue Y, Harris JR, Liang J, Noble PW, Hogan BL (2011). Multiple stromal populations contribute to pulmonary fibrosis without evidence for epithelial to mesenchymal transition. Proc Natl Acad Sci U S A.

[7] Barkauskas CE, Cronce MJ, Rackley CR, Bowie EJ, Keene DR, Stripp BR, Randell SH, Noble PW, Hogan BL (2013). Type 2 alveolar cells are stem cells in adult lung. J Clin Invest.

[8] Desai TJ, Brownfield DG, Krasnow MA (2014). Alveolar progenitor and stem cells in lung development, renewal and cancer. Nature.

[9] Tsuji T, Aoshiba K, Nagai A (2006). Alveolar cell senescence in patients with pulmonary emphysema. Am J Respir Crit Care Med.

[10] Xu X, Rock JR, Lu Y, Futtner C, Schwab B, Guinney J, Hogan BL, Onaitis MW (2012). Evidence for type II cells as cells of origin of K-Ras-induced distal lung adenocarcinoma. Proc Natl Acad Sci U S A.

[11] Sisson TH, Mendez M, Choi K, Subbotina N, Courey A, Cunningham A, Dave A, Engelhardt JF, Liu X, White ES (2010). Targeted injury of type II alveolar epithelial cells induces pulmonary fibrosis. Am J Respir Crit Care Med.

[12] Harrison JH, Porretta CP, Leming K (1995). Purification of murine pulmonary type II cells for flow cytometric cell cycle analysis. Exp Lung Res.

[13] Corti M, Brody AR, Harrison JH (1996). Isolation and primary culture of murine alveolar type II cells. Am J Respir Cell Mol Biol.

[14] Rice WR, Conkright JJ, Na CL, Ikegami M, Shannon JM, Weaver TE (2002). Maintenance of the mouse type II cell phenotype in vitro. Am J Physiol Lung Cell Mol Physiol.

[15] Lo B, Hansen S, Evans K, Heath JK, Wright JR (2008). Alveolar epithelial type II cells induce T cell tolerance to specific antigen. J Immunol.

[16] Teisanu RM, Chen H, Matsumoto K, McQualter JL, Potts E, Foster WM, Bertoncello I, Stripp BR (2011). Functional analysis of two distinct bronchiolar progenitors during lung injury and repair. Am J Respir Cell Mol Biol.

[17] Yamada M, Kubo H, Ota C, Takahashi T, Tando Y, Suzuki T, Fujino N, Makiguchi T, Takagi K, Suzuki T, Ichinose M (2013). The increase of microRNA-21 during lung fibrosis and its contribution to epithelial-mesenchymal transition in pulmonary epithelial cells. Respir Res.

[18] Cunningham AC, Milne DS, Wilkes J, Dark JH, Tetley TD, Kirby JA (1994). Constitutive expression of MHC and adhesion molecules by alveolar epithelial cells (type II pneumocytes) isolated from human lung and comparison with immunocytochemical findings. J Cell Sci.

[19] Harbeck RJ, Gegen NW, Struhar D, Mason R (1988). Class II molecules on rat alveolar type II epithelial cells. Cell Immunol.

[20] Debbabi H, Ghosh S, Kamath AB, Alt J, Demello DE, Dunsmore S, Behar SM (2005). Primary type II alveolar epithelial cells present microbial antigens to antigen-specific CD4+ T cells. Am J Physiol Lung Cell Mol Physiol.

[21] Kreisel D, Richardson SB, Li W, Lin X, Kornfeld CG, Sugimoto S, Hsieh CS, Gelman AE, Krupnick AS (2010). Cutting edge: MHC class II expression by pulmonary nonhematopoietic cells plays a critical role in controlling local inflammatory responses. J Immunol.

[22] Sato A, Yamada N, Ogawa Y, Ikegami M (2013). CCAAT/enhancer-binding protein-alpha suppresses lung tumor development in mice through the p38alpha MAP kinase pathway. PLoS One.

[23] King BA, Kingma PS (2011). Surfactant protein D deficiency increases lung injury during endotoxemia. Am J Respir Cell Mol Biol.

[24] Rawlins EL, Perl AK (2012). The a"MAZE"ing world of lung-specific transgenic mice. Am J Respir Cell Mol Biol.

[25] Lee JH, Kim J, Gludish D, Roach RR, Saunders AH, Barrios J, Woo AJ, Chen H, Conner DA, Fujiwara Y (2013). Surfactant protein-C chromatin-bound green fluorescence protein reporter mice reveal heterogeneity of surfactant protein C-expressing lung cells. Am J Respir Cell Mol Biol.

[26] Marsh LM, Cakarova L, Kwapiszewska G, von Wulffen W, Herold S, Seeger W, Lohmeyer J (2009). Surface expression of CD74 by type II alveolar epithelial cells: a potential mechanism for macrophage migration inhibitory factor-induced epithelial repair. Am J Physiol Lung Cell Mol Physiol.

[27] Assis DN, Leng L, Du X, Zhang CK, Grieb G, Merk M, Garcia AB, McCrann C, Chapiro J, Meinhardt A (2014). The role of macrophage migration inhibitory factor in autoimmune liver disease. Hepatology.

[28] Hood L, Steinmetz M, Malissen B (1983). Genes of the major histocompatibility complex of the mouse. Annu Rev Immunol.

[29] McQualter JL, Yuen K, Williams B, Bertoncello I (2010). Evidence of an epithelial stem/progenitor cell hierarchy in the adult mouse lung. Proc Natl Acad Sci U S A.

[30] O'Malley J, Matesic LE, Zink MC, Strandberg JD, Mooney ML, De Maio A, Reeves RH (1998). Comparison of acute endotoxin-induced lesions in a/J and C57BL/6J mice. J Hered.

[31] Meuwissen R, Berns A (2005). Mouse models for human lung cancer. Genes Dev.

[32] Kim CF, Jackson EL, Woolfenden AE, Lawrence S, Babar I, Vogel S, Crowley D, Bronson RT, Jacks T (2005). Identification of bronchioalveolar stem cells in normal lung and lung cancer. Cell.

[33] Herold S, von Wulffen W, Steinmueller M, Pleschka S, Kuziel WA, Mack M, Srivastava M, Seeger W, Maus UA, Lohmeyer J (2006). Alveolar epithelial cells direct monocyte transepithelial migration upon influenza virus infection: impact of chemokines and adhesion molecules. J Immunol.

[34] Messier EM, Mason RJ, Kosmider B (2012). Efficient and rapid isolation and purification of mouse alveolar type II epithelial cells. Exp Lung Res.

[35] Elizur A, Adair-Kirk TL, Kelley DG (2007). Griffin GL, deMello DE, senior RM: Clara cells impact the pulmonary innate immune response to LPS. Am J Physiol Lung Cell Mol Physiol.

[36] Elizur A, Adair-Kirk TL, Kelley DG, Griffin GL, Demello DE, Senior RM (2008). Tumor necrosis factor-alpha from macrophages enhances LPS-induced clara cell expression of keratinocyte-derived chemokine. Am J Respir Cell Mol Biol.

[37] Reutershan J, Morris MA, Burcin TL, Smith DF, Chang D, Saprito MS, Ley K (2006). Critical role of endothelial CXCR2 in LPS-induced neutrophil migration into the lung. J Clin Invest.

[38] Fujino N, Kubo H, Ota C, Suzuki T, Suzuki S, Yamada M, Takahashi T, He M, Suzuki T, Kondo T, Yamaya M (2012). A novel method for isolating individual cellular components from the adult human distal lung. Am J Respir Cell Mol Biol.

[39] Cunningham AC, Zhang JG, Moy JV, Ali S, Kirby JA (1997). A comparison of the antigen-presenting capabilities of class II MHC-expressing human lung epithelial and endothelial cells. Immunology.

[40] Autengruber A, Gereke M, Hansen G, Hennig C, Bruder D (2012). Impact of enzymatic tissue disintegration on the level of surface molecule expression and immune cell function. Eur J Microbiol Immunol (Bp).

