# Supplementary material for: Fraction of MHCII and EpCAM expression characterizes distal lung epithelial cells for alveolar type 2 cell isolation
Source: Respir Res. 2017 Aug 7;18:150. doi: 10.1186/s12931-017-0635-5 (PMC5545863; doi:10.1186/s12931-017-0635-5)
Supplement: Supplementary file 1 — An overview flowchart of cell isolation protocol. (PPTX 73 kb) [file 12931_2017_635_MOESM1_ESM.pptx]

## Slide 1
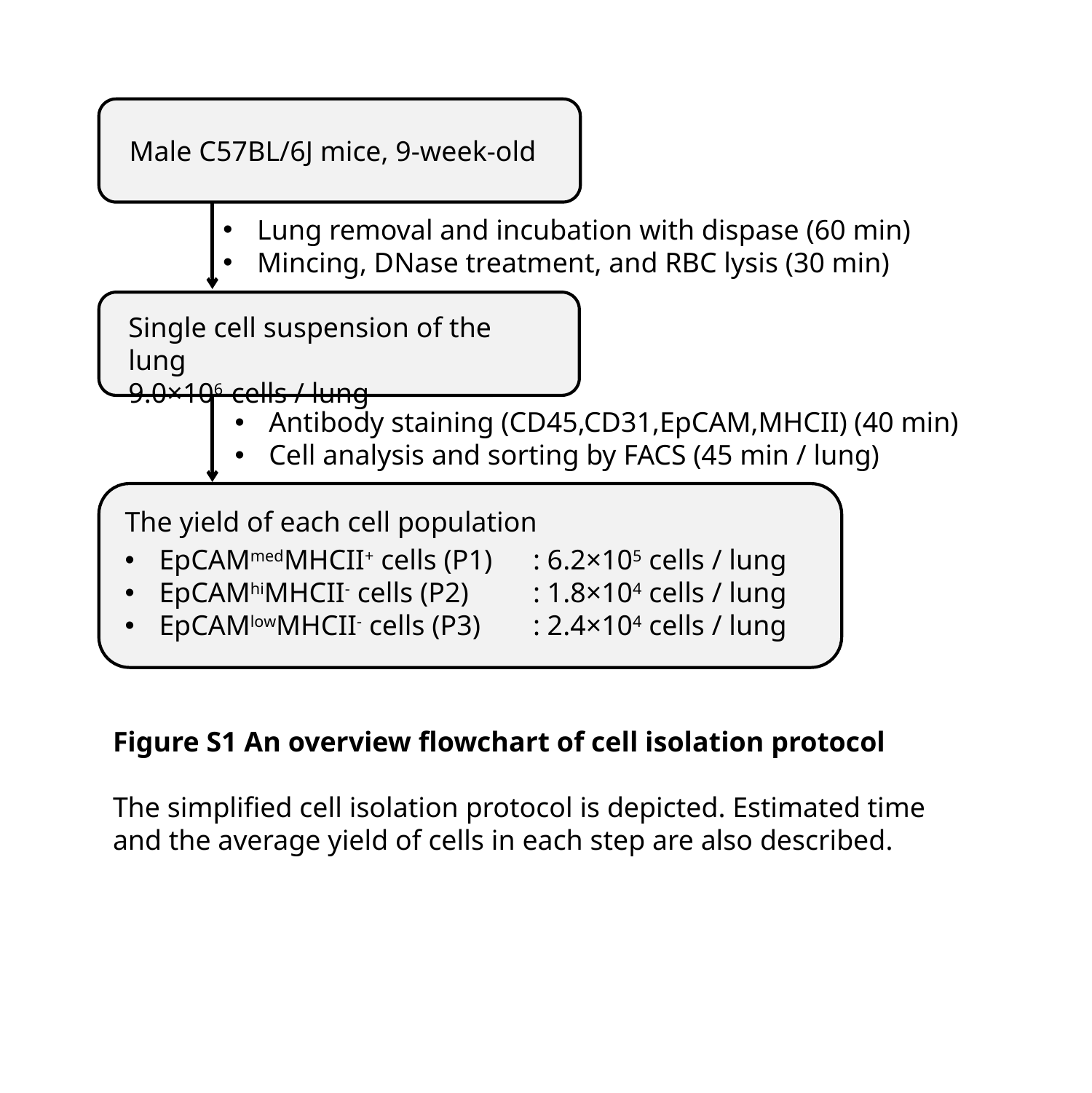

Male C57BL/6J mice, 9-week-old
Lung removal and incubation with dispase (60 min)
Mincing, DNase treatment, and RBC lysis (30 min)
Single cell suspension of the lung
9.0×10^6^ cells / lung
Antibody staining (CD45,CD31,EpCAM,MHCII) (40 min)
Cell analysis and sorting by FACS (45 min / lung)
The yield of each cell population
EpCAMmedMHCII+ cells (P1)
EpCAMhiMHCII- cells (P2)
EpCAMlowMHCII- cells (P3)
: 6.2×10^5^ cells / lung
: 1.8×10^4^ cells / lung
: 2.4×10^4^ cells / lung
Figure S1 An overview flowchart of cell isolation protocol
The simplified cell isolation protocol is depicted. Estimated time and the average yield of cells in each step are also described.
