# Supplementary material for: Fraction of MHCII and EpCAM expression characterizes distal lung epithelial cells for alveolar type 2 cell isolation
Source: Respir Res. 2017 Aug 7;18:150. doi: 10.1186/s12931-017-0635-5 (PMC5545863; doi:10.1186/s12931-017-0635-5)
Supplement: Supplementary file 2 — List of antibodies for flow cytometry. (DOCX 17 kb) [file 12931_2017_635_MOESM2_ESM.docx]

| Antibody | clone | Company | Catalog No. | Dilution | Isotype control |
| --- | --- | --- | --- | --- | --- |
| CD45-PE-Cy7 | 30-F11 | eBioscience | 25-0451-82 | 1:200 | Rat IgG2bκ-PE-Cy7 |
| CD31-PE-Cy7 | 390 | BD Biosciences | 561410 | 1:100 | Rat IgG2aκ-PE-Cy7 |
| EpCAM-APC | G8.8 | eBioscience | 17-5791-82 | 1:100 | Rat IgG2aκ-APC |
| MHCII-eFluor450 | I-A/I-E | eBioscience | 48-5321-82 | 1:150 | Rat IgG2bκ-eFluor450 |
| CD74-FITC | In-1 | BD Biosciences | 561491 | 1:200 | Rat IgG2bκ-FITC |
| proSP-C |  | Millipore | AB3796 | 1:400 | Rabbit IgG |
| EpCAM, epithelial cell adhesion molecule; MHCII, major histocompatibility complex II; proSP-C, pro-surfactant protein C; PE-Cy7, phycoerythrin-cyanine 7; APC, allophycocyanin; FITC, fluorescein isothiocyanate. | | | | | |

**Table S1: List of antibodies for flow cytometry**
