# Supplementary material for: Fraction of MHCII and EpCAM expression characterizes distal lung epithelial cells for alveolar type 2 cell isolation
Source: Respir Res. 2017 Aug 7;18:150. doi: 10.1186/s12931-017-0635-5 (PMC5545863; doi:10.1186/s12931-017-0635-5)
Supplement: Supplementary file 3 — Gating strategy for isolation of AT2 cells based on EpCAM and MHCII expression. (PPTX 301 kb) [file 12931_2017_635_MOESM3_ESM.pptx]

## Slide 1
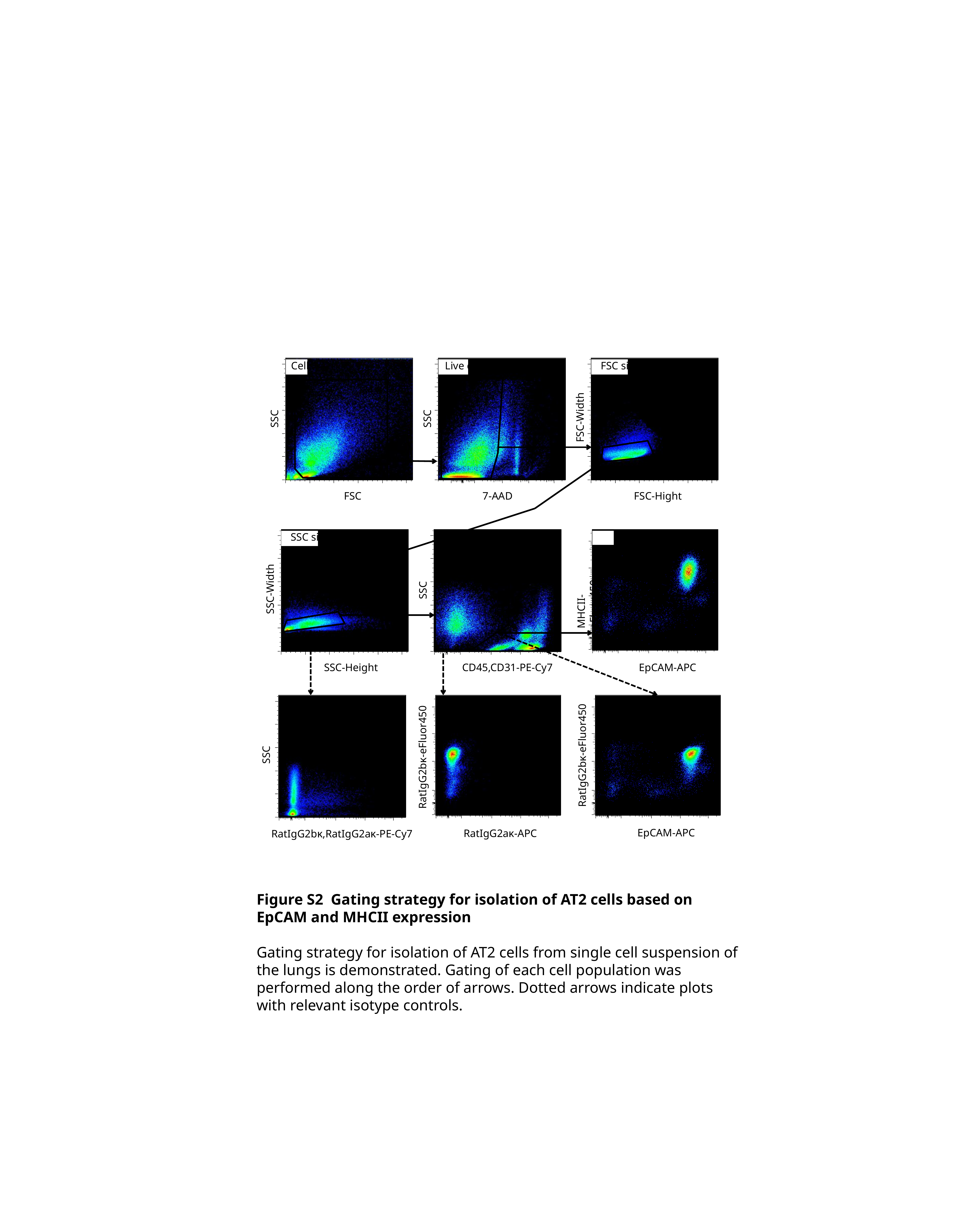

Cells
84.8
SSC
FSC
Live cells
86.6
SSC
7-AAD
FSC singlet
FSC-Width
95.5
FSC-Hight
SSC singlet
SSC-Width
92.0
SSC-Height
CD45-CD31- cells
28.1
SSC
CD45,CD31-PE-Cy7
P1
89.3
MHCII-eFluor450
1.6
P2
2.6
P3
EpCAM-APC
SSC
RatIgG2bκ,RatIgG2aκ-PE-Cy7
RatIgG2bκ-eFluor450
RatIgG2aκ-APC
RatIgG2bκ-eFluor450
EpCAM-APC
Figure S2 Gating strategy for isolation of AT2 cells based on EpCAM and MHCII expression
Gating strategy for isolation of AT2 cells from single cell suspension of the lungs is demonstrated. Gating of each cell population was performed along the order of arrows. Dotted arrows indicate plots with relevant isotype controls.
