# Supplementary material for: Fraction of MHCII and EpCAM expression characterizes distal lung epithelial cells for alveolar type 2 cell isolation
Source: Respir Res. 2017 Aug 7;18:150. doi: 10.1186/s12931-017-0635-5 (PMC5545863; doi:10.1186/s12931-017-0635-5)
Supplement: Supplementary file 4 — The applicability of the AT2 isolation strategy to different strains and ages of mice. (PPTX 572 kb) [file 12931_2017_635_MOESM4_ESM.pptx]

## Slide 1
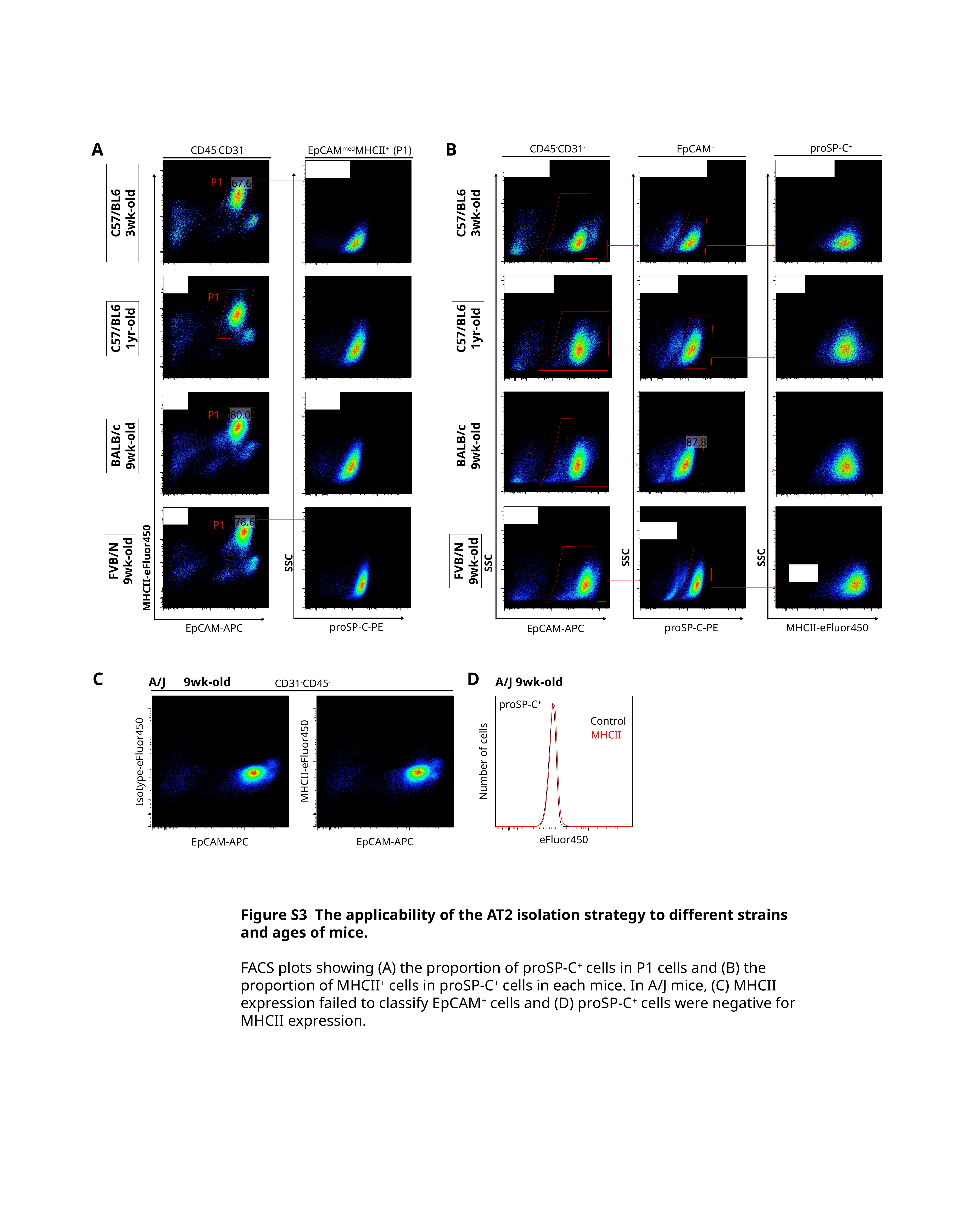

A
B
proSP-C+
CD45-CD31-
EpCAM+
CD45-CD31-
EpCAMmedMHCII+ (P1)
81.3
82.8
0.5
99.5
67.6
97.5
2.5
P1
C57/BL6
3wk-old
C57/BL6
3wk-old
90.5
84.4
0.9
99.1
78.2
97.0
3.00
P1
C57/BL6
1yr-old
C57/BL6
1yr-old
92.5
87.8
99.1
0.9
80.0
97.2
2.8
P1
BALB/c
9wk-old
BALB/c
9wk-old
93.4
85.9
0.3
99.7
78.6
98.6
1.4
P1
FVB/N
9wk-old
FVB/N
9wk-old
SSC
SSC
SSC
SSC
MHCII-eFluor450
proSP-C-PE
proSP-C-PE
MHCII-eFluor450
EpCAM-APC
EpCAM-APC
C
D
A/J　9wk-old
A/J 9wk-old
CD31-CD45-
Isotype-eFluor450
EpCAM-APC
MHCII-eFluor450
EpCAM-APC
Number of cells
eFluor450
proSP-C+
(Control)
(MHCII)
Control
MHCII
Figure S3 The applicability of the AT2 isolation strategy to different strains and ages of mice.
FACS plots showing (A) the proportion of proSP-C+ cells in P1 cells and (B) the proportion of MHCII+ cells in proSP-C+ cells in each mice. In A/J mice, (C) MHCII expression failed to classify EpCAM+ cells and (D) proSP-C+ cells were negative for MHCII expression.
