# Supplementary material for: Fraction of MHCII and EpCAM expression characterizes distal lung epithelial cells for alveolar type 2 cell isolation
Source: Respir Res. 2017 Aug 7;18:150. doi: 10.1186/s12931-017-0635-5 (PMC5545863; doi:10.1186/s12931-017-0635-5)
Supplement: Supplementary file 5 — The yield of single cell suspension and the proportion of each cell population in control and LPS-induced injury mice. (DOCX 14 kb) [file 12931_2017_635_MOESM5_ESM.docx]

**Table S2: The yield of single cell suspension and the proportion of each cell population in control and LPS-induced injury mice.**

|  | The yield of single cell suspension  (×10^6^/ lung) | CD45^-^CD31^-^ cells  /Live single cells  (%) | P1 cells  /CD45^-^CD31^-^ cells  (%) | P2 cells  /CD45^-^CD31^-^ cells  (%) | P3 cells  /CD45^-^CD31^-^ cells  (%) |
| --- | --- | --- | --- | --- | --- |
| PBS group | 8.3±0.8 | 38.3±1.7 | 83.4±0.9 | 1.5±0.1 | 5.5±0.4 |
| LPS group | 22.0±2.1 | 17.8±0.9 | 76.4±3.5 | 2.9±0.5 | 5.7±1.0 |
| LPS, lipopolysaccharide. | | | | | |
